# Fractionated stereotactic radiotherapy of brainstem metastases – Clinical outcome and prognostic factors

**DOI:** 10.1016/j.ctro.2024.100893

**Published:** 2024-11-21

**Authors:** Anna Krämer, Laura Hahnemann, Fabian Schunn, Christoph A. Grott, Michael Thomas, Petros Christopoulos, Jonathan W. Lischalk, Juliane Hörner-Rieber, Philipp Hoegen-Saßmannshausen, Tanja Eichkorn, Maximilian Y. Deng, Eva Meixner, Kristin Lang, Angela Paul, Fabian Weykamp, Jürgen Debus, Laila König

**Affiliations:** a Department of Radiation Oncology, University Hospital of Heidelberg, Im Neuenheimer Feld 400, 69120 Heidelberg, Germany; b National Center for Radiation Oncology (NCRO), Heidelberg Institute for Radiation Oncology (HIRO), Im Neuenheimer Feld 400, 69120 Heidelberg, Germany; c National Center for Tumor Diseases (NCT) Heidelberg, Im Neuenheimer Feld 400, 69120 Heidelberg, Germany; d Department of Thoracic Oncology, Thoraxklinik and National Center for Tumor Diseases at Heidelberg University Hospital, Heidelberg, Germany; e Translational Lung Research Center Heidelberg (TLRC-H), Member of the German Center for Lung Research (DZL), Germany; f Department of Radiation Oncology, Perlmutter Cancer Center at New York University Langone Health at Long Island, New York, NY, USA; g Clinical Cooperation Unit Radiation Oncology (E050), German Cancer Research Center (DKFZ), Im Neuenheimer Feld 280, 69120 Heidelberg, Germany; h Heavy Ion Therapy Center (HIT), Heidelberg University Hospital, Im Neuenheimer Feld 450, 69120 Heidelberg, Germany

**Keywords:** Fractionated stereotactic radiotherapy, CyberKnife, Brainstem metastases, Radiation-induced contrast enhancement, Immunotherapy

## Abstract

**Introduction:**

Brain metastases (BM) are the most common malignancy in the central nervous system (CNS) and observed in approximately 30% of cancer patients. Brainstem metastases (BSM) are challenging because of their location and the associated neurological risks. There are still no general therapeutic recommendations in this setting. Stereotactic radiosurgery (SRS) is one of few possible local therapy options but limited due to the tolerance dose of the brainstem. There is still no standard regarding the optimal dose und fractionation.

**Methods:**

We retrospectively analyzed 65 patients with fractionated stereotactic radiotherapy (fSRT) for 69 BSM. FSRT was delivered at a dose of 30 Gy in six fractions prescribed to the 70 % isodose performed with Cyberknife. Overall survival (OS), local control (LC) and total intracranial brain control (TIBC) were analyzed via Kaplan-Meier method. Cox proportional hazards models were used to identify prognostic factors.

**Results:**

Median follow-up was 27.3 months. One-year TIBC was 35.0 % and one-year LC was 84.1 %. Median OS was 8.9 months. In total, local progression occurred in 7.7 % and in 8.2 % symptomatic radiation-induced contrast enhancements (RICE) were diagnosed. In univariate analysis the Karnofsky performance scale index (KPI) (p = 0,001) was an independent prognostic factor for longer OS. Acute CTCAE grade 3 toxicities occurred in 18.4 %.

**Conclusion:**

FSRT for BSM is as an effective and safe treatment approach with high LC rates and reasonable neurological toxicity despite the poor prognosis in this patient cohort is still very poor. Clinical and imaging follow-up is necessary to identify cerebral progression and adverse toxicity including RICE.

## Highlights

• Stereotactic radiotherapy with 30 Gy in 6 fractions @70 % isodose for BSM is effective.
• There was an excellent local control rate with local progression only in 7.7%.
• Treatment-related toxicity was rare and mostly with moderate symptoms.
• Long term clinical and radiological follow-up is essential.

## 1 Introduction

Brain metastases (BM) are the most common diagnosed malignancy in the central nervous system (CNS). Cancer patients have a risk of about 30 % for developing BM during their course of disease [1], [2]. The risk of developing BM increase with the primary tumor histology and more advanced tumor stage [3], [4]. The effect of systemic therapy in the treatment of BM is significantly reduced due to the blood–brain barrier [5]. However the development of immunotherapies (IT), target therapies (TT), and the understanding of the treatment effects and the microenvironment has resulted in improved survival in a subgroup of patients with driver mutations [6], [7], [8], [9], [10].

Stereotactic radiosurgery (SRS) is a well-established treatment option which has become a frequently used treatment for up to ten BM due to the excellent local tumor control of 80–90 % [11], [12], and a superior toxicity profile compared to whole brain radiotherapy (WBRT) with significantly less deterioration in memory and learning function [13]. So far, the associations between a combination of SRS and immunotherapies (IT) and the occurrence of radiation-induced contrast enhancement (RICE) have not been elucidated [14]. Due to the risk of serious side effects combination treatment is used cautiously. To avoid equating treatment effect with real tumor progression, the Response Assessment in Neuro-Oncology (RANO) criteria are used as standardized guideline [15], [16]. The DEGRO practical guideline has defined a multistep approach for distinguishing between blood–brain barrier disruptions (BBD) – also referred to as “pseudoprogression” – and radiation-induced necrosis (RN). An interdisciplinary consultation is necessary to establish the diagnosis and to determine the right therapy because not all RICE require intervention [17]. Radiotherapy combined with systemic therapy show high efficacy depending on tumor entity and active ingredient [18], [19], [20]. Nevertheless, there is concern about more severe side effects, especially with stereotactic radiation. The combination of IT and TT appears to be a treatment factor associated with the development of RICE. However, the clinical evidence is still limited and prospective studies are lacking [21], [22].

Due to their critical localization of BSM they often yield severe neurological complications and surgical intervention is not possible due to the high risk of morbidity and mortality [23], [24], [25]. BSM have an incidence of approximate 1–7 % of all BM [23], [26], [27]. There are no prospective randomized studies and therefore no general therapeutic recommendations can be provided. Patients with BSM are often excluded from studies because of their expected poor prognosis and the therapeutic challenge. Prior retrospective investigations have shown SRS of BSM to be safe with tolerable toxicity and excellent local control (LC) up to 90 % after one year, depending on the tumor volume [25], [28], [29], [30], [31], [32]. Other prognostic factors include the number of BM, the Karnofsky Performance Status (KPS), controlled primary tumor and the tumor histology [32], [33]. Furthermore, SRS of BSM frequently enables improvement in neurological symptoms and treatment is only rarely associated with high grade toxicity [25], [26], [29], [30]. A previously delivered WBRT increases the risk of side effects further [24], [31]. Due to the lack of therapeutic guidelines, the fractionation and the prescribed dose of radiotherapy (RT) depends on the institution [24]. The limiting factor is the tolerance dose of the brainstem: In conventionally fractionated radiotherapy the tolerable maximum dose of the whole brainstem is approximately 54 Gray (Gy). In the case of SRS of BSM a single dose of 12 Gy should not be exceeded [34], [35]. Fractionated RT is used to reduce toxicity, for example < 23,1 Gy in three fractions or < 31 Gy in five fractions [1]. Leemann et al. showed that LC improved significantly improved with doses over 20 Gy delivered in 1–5 fractions, mostly in one (56 %) or three (36 %) fractions with fractionation leading to superior toxicity. However, the patient sample was limited [36].

There are still no therapeutic guidelines for the radiotherapy of BSM particularly in the combination with new targeted therapies. We therefore aimed to present clinical outcomes from patients treated with fractionated stereotactic radiotherapy (fSRT) for BSM. Additionally, the aim was to investigate possible interactions with sequential or simultaneous IT/TT and the risk of RICE.

## 2 Methods

### 2.1 Patient selection

In this study, all patients were treated at a European tertiary cancer center between April 2016 and December 2021 and were available for analysis. Only patients who received fSRT to a BSM treated with 30 Gy in 6 fractions prescribed to the 70 % isodose were included in this analysis. Patient, treatment, and survival data were obtained from the clinical research database, the nation cancer database and from review of the medical records. The analysis was performed retrospectively and longitudinally throughout the follow-up period. This study was approved by the institutional ethics committee (S-494/2021).

### 2.2 Treatment

Oncologic treatment recommendations were made in an interdisciplinary setting and fSRT was performed with a CyberKnife M6 (Accuray Inc., Sunnyvale, California) system. For head immobilization, a custom thermoplastic mask was shaped. The planning scans for fSRT treatment included computed tomography (CT) and high-resolution magnetic resonance imaging (MRI). Dose constraints in nontarget/normal tissue followed the recommendation of the UK Consensus on Normal Tissue Dose Constraints for Stereotactic Radiotherapy and the October 2008 issue of Seminars in Radiation Oncology [37], [38], [39]. Target volume was generated in accordance with the established institutional guidelines: (1) the contrast-enhanced tissue on the T1-weighted image was contoured as gross tumor volume (GTV). By adding (2) an isotopic margin of 1 mm the planning target volume (PTV) was generated. Following the department’s protocol, 30 Gy was prescribed in 6 fractions to the surrounding 70 % isodose for all patients in this study. Patients received a daily 4 mg dose of dexamethasone as prophylaxis for edema during treatment.

Any dose of systemic therapies administered within 14 days before or after fSRT was recorded and defined as simultaneous.

### 2.3 Follow-up

Post-fSRT follow-up consisted of clinical-neurological examination and contrast enhanced brain MRI. Patients were seen at regular three-months intervals or as clinically required. Any lesions suspicious of distant progression were reviewed by an interdisciplinary team. Salvage WBRT was recommended for patients with multiple (≥10) distant lesions and/or leptomeningeal disease progression (LMD).

### 2.4 Outcome

OS was calculated from the first day of fSRT to the date of death or to the date of last contact in clinic. TIBS and LC was defined as time between the start of fSRT and the diagnosis of intracranial/ local progression based on MRI findings or the date of the last available MRI without progression.

Based on the BM response and progression criteria proposed by the Response Assessment in Neuro-Oncology (RANO) group local or distant progressive BM were determined. Lin et al. already pointed out that posttreatment imaging should be interpreted with caution. Determining whether contrast enhancement is due to tumor progression or to treatment-associated effects such as RN can be difficult. Moreover, the term of RN itself is not consistent used throughout the literature and therefore homogeneous diagnosis can be challenging [15]. In this analyse, we used the term “RICE” with every clinically evident RICE lesion suspicious of RN. Those lesions were reviewed in an interdisciplinary tumor board and Diagnosis was made using a multistep approach as recommended in the guideline for central nervous system radiation necrosis by the Deutsche Gesellschaft für Radioonkologie (DEGRO) [17].

Acute (during treatment until 3 months after fSRT) and chronic (>3 months after fSRT) toxic effects were assessed during follow-up. Toxicity was graded according to the Common Terminology Criteria for Adverse Events (CTCAE), version 5.0.

### 2.5 Statistical analyses

The statistical analysis methods used are comparable to the recently published study about fSRT of intracranial postoperative cavities [40].

Descriptive statistics were used for baseline parameters. Continuous variables are given as median (with range or Q1Q3) values and categorial variables as absolute or relative frequencies. Kaplan-Meier analysis was performed to estimate OS, TIBS and LC. Univariate cox regression analysis was performed on potential prognostic factors. Due to the low number of cases, cox proportional hazard analyses were not fitted for LC nor for RICE. RICE statistics were analyzed with chi-square test. P-value ≤ 0.05 was considered to indicate a statistical significance. All statistical analyses were carried out with SPSS (version 28; SPSS, Chicago, IL).

## 3 Results

In this study, 65 patients with a total of 186 treatment sessions on 369 lesions including 69 BSM were retrospectively analyzed.

### 3.1 Patient/ treatment characteristics

The median patient age at fSRT was 64 years (range: 25–87 years). Lung cancer (n = 38; 58.5 %), breast cancer (n = 11; 16.9 %) and melanoma (n = 8; 12.3 %) were the most frequent primary. Of the patients with non-small cell lung cancer, 2 out of 27 had targeted mutations like EGFR (n = 1) or ALK (n = 1) mutations and 15 tumors were PD-L1 positive. A total of 24 (36.9 %) patients had BM at primary diagnosis. More than two thirds of the patients had uncontrolled extracranial disease (n = 44; 67.7 %) and extracranial metastasis (n = 53; 81.5 %). The median KPS was 80 % (Q1-Q3: 70 %-85 %). Detailed patient characteristics are illustrated in Table 1.

**Table 1:** Patient Characteristics.

**Characteristic**		**n = 65**	**%**
**Patients/BSM**		65/69	
**Gender**			
	male	33	50.8
	female	32	49.2
**Age at fSRT on BSM, y**			
	median (min–max)	64 (25–87)	
**Primary cancer site**			
	adeno-NSCLC	27	41.5
	breast cancer	11	16.9
	melanoma	8	12.3
	non-adeno-NSCLC	4	6.2
	SCLC	4	6.2
	others	11	17.4
**Initial BM**			
	yes	24	36.9
	no	41	63.1
**KPS at fSRT**			
	Median (Q1-Q3)	80 (70–85)	
**KPS stratified**			
	90–100	16	24.6
	70–80	43	66.2
	≤60	6	9.2
**Metastases outside CNS at fSRT**			
	yes	53	81.5
	no	12	18.5
**Extracranial disease at fSRT**			
	controlled	21	32.3
	not controlled	44	67.7
**IT/TT simultaneous to fSRT^a^**			
	yes	20	30.8
	no	45	69.2
**CTx simultaneous to fSRT^b^**			
	yes	11	16.9
	no	54	83.1

**Abbreviation:** BSM, brain stem metastases; fSRT, fractionated stereotactic radiotherapy; y, years; NSCLC, non-small-cell lung cancer; SCLC, small-cell lung cancer; BM, brain metastases; KPS, Karnofsky Performance Status; CNS, central nervous system; IT, immunotherapy; TT, targeted therapy; CTx, chemotherapy. ^a^ Any dose of immunotherapies given within 14 days before or after fSRT ^b^ Any dose of chemotherapies given within 14 days before or after fSRT.

20 (30.8 %) patients received simultaneous IT/TT, 11 (16.9 %) patients received simultaneous CTx and 7 patients (10.8 %) received both. Supplementary Tables 1 and 2 are detailing the distribution of IT/TT and CTx.

The median PTV volume of the BSM was 0.59 ml (Q1-Q3: 0.22–2.09 ml). Median fSRT time was 29 min (Q1-Q3: 24–40 min). Details on treatment characteristics are listed in Table 2.

**Table 2:** Treatment and Radiotherapy Characteristics.

**Characteristic**		**n = 65**	**%**
**Prior fSRT/SRS on other BM**			
	yes	18	27.7
	no	47	72.3
**Combined fSRT/SRS on additional BM**			
	yes	43	66.2
	no	22	33.8
**Prior WBRT**			
	yes	7	10.8
	no	58	89.2
**Collimator type**			
	fix	53	81.5
	IRIS	12	18.5
**fSRT time, min**			
	median (Q1-Q3)	29 (24–40)	
**PTV volume BSM, ml**			
	median (Q1-Q3)	0.59 (0.22–2.09)	

**Abbreviation:** fSRT, fractionated stereotactic radiotherapy; SRS, stereotactic radiosurgery; BM, brain metastases; WBRT, whole brain radiotherapy; min, minutes; PTV, planning target volume; ml, milliliters.

### 3.2 Oncological endpoints

Using the reverse Kaplan-Meier method, the median follow-up for OS was 27.3 months (95 %-CI: 20.8–33.8). Relative frequencies of progression and RICE are related to a total of 49 patients. 16 patients were not included in this analysis, because they did not receive follow-up MRI or results were not possible to analyze.

**Fig. 1:**
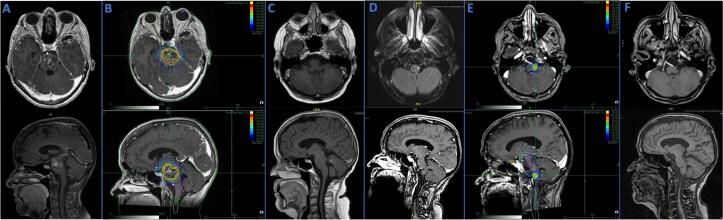
Case example with MRI findings and treatment plans. This case example is about a female patient with HER2 positiv breast cancer initially diagnosed in 2014. In Dezember 2016 this patient experiened paralysis and dumbness on the right body side. A Axial and sagital view, T1 MPRAGE weighted MRI (05/2017). Brainstem metastasis located in the pons with cental necrosis and perifocal edema, 2.6 x 2.0 cm. B Treatment plan for fSRT at the CyberKnife with 30 Gy in 6 fractions at 70 % isodose performed with the CyberKnife, PTV 15.27 ml (05/2017). C Axial and sagital view, T1-weighted MRI (10/2017. The brainstem metastasis showed already a good treatment response 5 months after fSRT. D Axial view,T2-Flair and sagital view T1-MPRAGE weighted MRI (08/2021). After four years new brainstem metastasis located in the medulla oblongata left, 0.2 x 0.2 cm. E Treatment plan for fSRT at the CyberKnife with 30 Gy in 6 fractions at 70 % isodose performed with the CyberKnife, PTV 0.21 ml (08/2021). F Axial view T1 + KM and sagital view T1 MPRAGE weighted MRI (11/2021). Treatment response in the brainstem after three months.

**Fig. 2:**
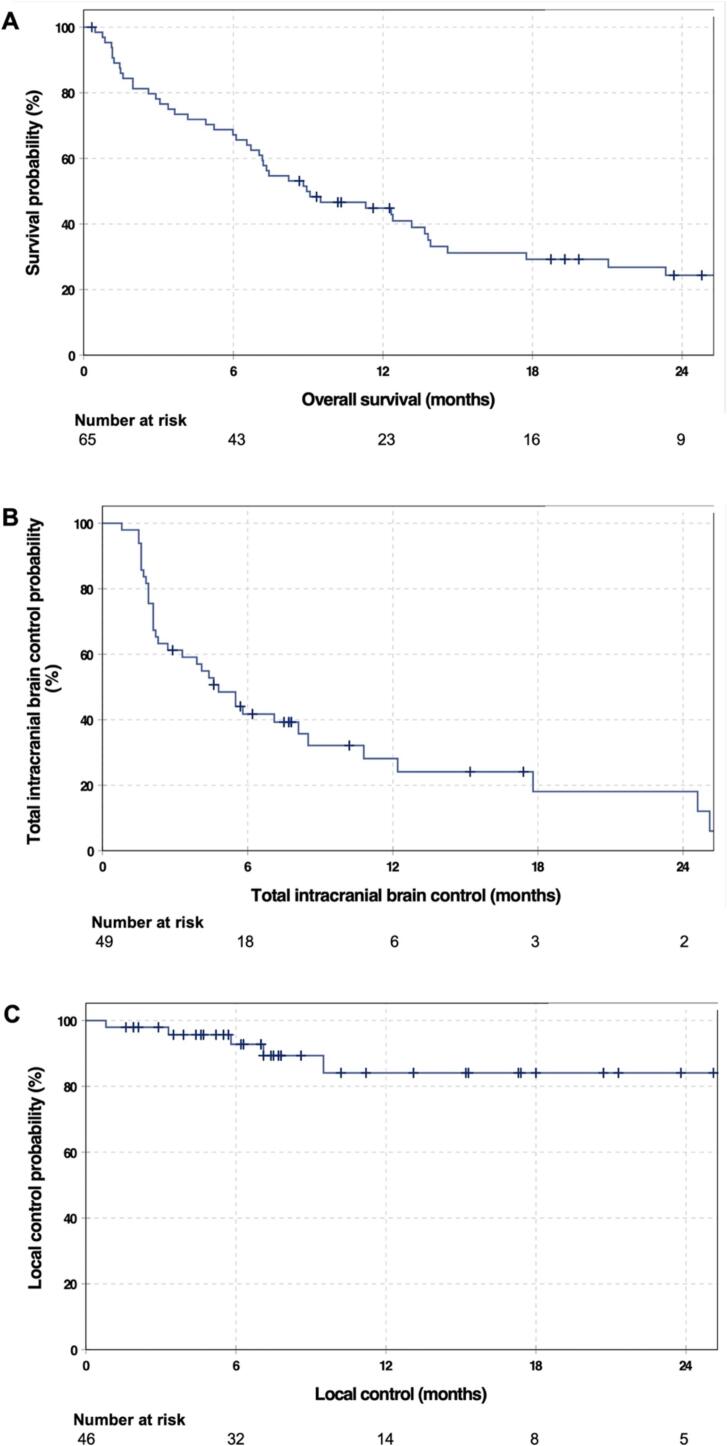
Kaplan-Meier-Curves. **A** Overall survival with corresponding numbers at risk. The median overall survival in the cohort (n = 65) was 8.9 months (95 %-CI: 4.6–13.2). **B** Total intracranial brain control with corresponding numbers at risk. The median total intracranial brain control in the cohort (n = 49) was 5.5 months (95 %-CI 0.8–10.2). **C** Local control with corresponding numbers at risk. The median local control in the cohort (n = 49) was not reached yet.

Thirty-six patients (73.5 %) developed CNS progression after BSM fSRT, of which 31 (86.1 %) had a distant cerebral progression, two (5.6 %) had a local progression and three (8.3 %) had both, local and distant progression. Fig. 1 illustrates a case example of a patient treated with FSRT. The median TIBC was 5.5 months (95 %-CI: 0.8–10.2 months). Median LC was not reached yet. LC at six months, one and two years was 92.8 %, 84.1 % and 84.1 % respectively. Kaplan Meier curves of TIBC and LC are shown in Fig. 2.

10 (15.4 %) patients had received a salvage WBRT at a median time of 6.2 months (Q1-Q3: 2.5–8.4 months) after fSRT (Table 3).

**Table 3:** Outcomes and Radiation necrosis.

**Characteristic**		**n = 65/49^a^**	**%**
A) **Outcomes**			
**CNS progression**			
**no**		13	20.0
**yes**		36	55.4
	distant	31	86.1
	local	2	5.6
	distant + local	3	8.3
**no follow-up image**		16	24.6
**Number of progressive BM**			
	median (Q1-Q3)	4 (2–6)	
	multiple^b,c^	14	38.9
**Further fSRT/SRS of BM elsewhere after fSRT on BSM^b^**			
	yes	21	42.9
	no	28	57.1
**Time from fSRT to next fSRT/SRS, mo**			
	median (Q1-Q3)	3.0 (2.3–6.4)	
**Salvage-WBRT**			
	yes	10	15.4
	no	55	84.6
**Time from fSRT to Salvage-WBRT, mo**			
	median (Q1-Q3)	6.2 (2.5–8.4)	
**Neurological death**			
	likely	11	16.9
	Not likely or alive at last contact	38	58.5
	no clinical information	16	24.6
B) **Radiation-induced contrast enhancement**			
**RICE on any BM during follow-up**			
	yes	17	26.2
	no	32	49.2
	no follow-up image	16	24.6
**RICE localization^b^**			
	brain stem	4	8.2
	other radiated BM	13	26.5
**Time from fSRT to RICE in brain stem, mo**			
	Median (Q1-Q3)	3.2 (1.7–6.2)	
**Clinical RICE symptoms (CTCAE grading), n = 4**			
	Grade 1	0	0.0
	Grade 2	1	25.0
	Grade 3	0	0.0
	Grade 4	3	75.0
	Grade 5	0	0.0
**Therapy for RICE in brain stem, n = 4**			
	no intervention	0	0.0
	Dexa only	1	25.0
	Dexa + Bevacizumab	3	75.0
**IT/TT simultaneous to fSRT^d^, n = 4**			
	yes	0	0.0
	no	4	100.0
**CTx simultaneous to RICE^e^, n = 4**			
	yes	1	25.0
	no	3	75.0

**Abbreviation:** CNS, central nervous system; BM, brain metastases; fSRT, fractionated stereotactic radiotherapy; SRS, stereotactic radiosurgery; BSM brain stem metastases; mo, months; WBRT, whole brain radiotherapy; RICE, radiation-induced contrast enhancement; CTCAE, Common Terminology Criteria for Adverse Events; Dexa, Dexamethason; IT, immunotherapy; TT, targeted therapy; CTx, chemotherapy. ^a^65 patients were included into the survival analysis, 16 patients did not have follow-up MRI and therefore are not included into progression analysis. ^b^ Relative frequencies are related to the 49 patients with follow-up MRI. ^c^ ≥ 10 progressive brain metastases. ^d^ Any dose of immunotherapies given within 14 days before or after fSRT. ^e^ Any dose of chemotherapies given within 14 days before or after diagnosis of RICE.

During follow-up, 47 of the 65 patients (72.3 %) had died – 27 of them (41.5 %) most likely due to neurological causes. The median OS was 8.9 months (95 %-CI: 4.6–13.2 months). The OS at six months, one and two years were 67.2 %, 44.8 % and 24.4 %. A Kaplan-Meier curve for OS of the study cohort is displayed in Fig. 1.

### 3.3 Prognostic factors

In univariate analysis, a higher KPS at the time of fSRT was predictive for superior OS (HR 0.960; 95 %-CI 0.936–0.984; p = 0.001). Therapy with IT/TT during course of disease (HR 0. 828; 95 %-CI 0.838–4.228; p = 0.524) or simultaneously to fSRT on BSM (HR 0.666; 95 %-CI 0.351–1.265; p = 0.175) showed a tendency to superior OS, but this result was not significant.

For TIBC, cox regression analyses revealed KPS ≥ 80 % at time of fSRT being significantly associated with superior TIBC (p = 0.028). Further outcomes of univariate cox regression are listed in Table 4, Table 5..

**Table 4:** Analyzed Factors in Univariate Cox Regression with the corresponding HR and P-values for Overall Survival.

**Factor analyzed**	**HR**	**95 % CI**	**P-value**
**Univariate Cox Regression**			
Gender (female* vs. male)	1.037	0.584–1.841	0.901
IT/TT during course of disease (yes* vs. no)	0.828	0.838–4.228	0.524
IT/TT simultaneous to fSRT^a^ (yes* vs. no)	0.666	0.351–1.265	0.214
CTx during course of disease (yes* vs. no)	1.272	0.642–2.520	0.490
CTx simultaneous to fSRT^b^ (yes* vs. no)	0.873	0.389–1.961	0.742
Initial BM (yes* vs. no)	0.772	0.417–1.426	0.408
Age at fSRT on BSM, y	1.015	0.987–1.044	0.303
Age stratified (≥64* vs. < 64)	1.425	0.797–2.547	0.232
Extracranial disease at fSRT (controlled* vs. uncontrolled)	0.683	0.367–1.270	0.223
Absent Extracranial Metastases at fSRT (yes* vs. no)	0.531	0.237–1.194	0.126
KPS at fSRT on BSM	0.960	0.936–0.984	**0.001**
KPS stratified (≥80 %* vs. < 80 %)	0.335	0.181–0.621	**<0,001**
Prior fSRT/SRS (yes* vs. no)	0.513	0.263–1.002	*0.051*
Combined fSRT/SRS on other BM (yes* vs. no)	1.205	0.655–2.217	0.549
RT time	1.016	0.996–1.037	0.107
PTV volume BSM, ml	0.988	0.897–1.089	0.810
PTV volume stratified, ml (≥0.59* vs. < 0.59)	1.540	0.857–2.768	0.149

**Abbreviation:** HR, hazard ratio; CI, confidence interval; vs, versus; IT, immunotherapy; TT, targeted therapy; fSRT, fractionated stereotactic radiotherapy; CTx, chemotherapy; BM, brain metastases; y, years; CNS, central nervous system; KPS, Karnofsky Performance Status; SRS, stereotactic radiosurgery; BSM, brainstem metastasis; RT, radiotherapy; PTV, planning target volume; ml, milliliters. ^a^ Any dose of immunotherapies given within 14 days before or after fSRT. ^b^ Any dose of chemotherapies given within 14 days before or after fSRT.

**Table 5:** Analyzed Factors in Univariate Cox Regression with the corresponding HR and P-values for Total Intracranial Brain Control.

**Factor analyzed**	**HR**	**95 % CI**	**P-value**
**Univariate Cox Regression**			
Gender (female* vs. male)	1.449	0.742–2.829	0.278
IT/TT during course of disease (yes* vs. no)	0.828	0.838–4.228	0.524
IT/TT simultaneous to fSRT^a^ (yes* vs. no)	1.046	0.528–2.071	0.898
CTx during course of disease (yes* vs. no)	1.135	0.510–2.526	0.756
CTx simultaneous to fSRT^b^ (yes* vs. no)	0.879	0.381–2.024	0.761
Initial BM (yes* vs. no)	0.506	0.242–1.060	0.071
Age at fSRT on resection cavity, y	1.004	0.975–1.034	0.809
Age stratified (≥64* vs. < 64)	1.025	0.523–2.010	0.942
Extracranial disease at fSRT (controlled* vs. uncontrolled)	0.797	0.396–1.606	0.526
Absent Extracranial Metastases at fSRT (yes* vs. no)	0.686	0.284–1.659	0.403
KPS at fSRT on BSM	0.647	0.297–1.412	0.274
KPS stratified (≥80 %* vs. < 80 %)	0.432	0.204–0.913	**0.028**
Prior fSRT/SRS (yes* vs. no)	0.773	0.384–1.556	0.470
Combined fSRT/SRS on other BM (yes* vs. no)	1.263	0.640–2.493	0.500
RT time	1.012	0.985–1.039	0.398
PTV volume BSM, ml	0.973	0.877–1.081	0.611
PTV volume stratified, ml (≥0.59* vs. < 0.59)	0.892	0.449–1.773	0.745

**Abbreviation:** HR, hazard ratio; CI, confidence interval; vs, versus; IT, immunotherapy; TT, targeted therapy; fSRT, fractionated stereotactic radiotherapy; CTx, chemotherapy; BM, brain metastases; y, years; CNS, central nervous system; KPS, Karnofsky Performance Status; BSM, brainstem metastasis; SRS, stereotactic radiosurgery; RT, radiotherapy; PTV, planning target volume; ml, milliliters; ^a^ Any dose of immunotherapies given within 3 months before or after fSRT. ^b^ Any dose of chemotherapies given within 3 months before or after fSRT. ^c^ Cumulative PTV volume of all brain metastases treated with fSRT/SRS per patient.

### 3.4 Toxicity

Acute and chronic treatment associated side effects were evaluable for 49 and 39 patients respectively. Most frequent acute side effects were grade 1–2 fatigue (41 % and 6 %), ataxia (12 % and 2 %) and headache (16 %). Reported acute grade 3 side effects were cerebral edema (2 %), seizure (4 %), headache (2 %) and cognitive impairment (2 %). Long term grade 3 toxicity were ataxia (8 %), headache (3 %) and nausea (3 %). Detailed acute and chronic adverse events data are provided in Table 6.

**Table 6:** Acute and Chronic Adverse Events.

	Acute^a^ (n = 49)					Chronic^b^ (n = 39)				
	*Grade 1*	*Grade 2*	*Grade 3*	*Grade 4*	*Grade 5*	*Grade 1*	*Grade 2*	*Grade 3*	*Grade 4*	*Grade 5*
Alopecia	2 (4 %)	1 (2 %)	−	−	−	1 (3 %)	0	−	−	−
Amnesia	0	0	0	−	−	0	1 (3 %)	0	−	−
Ataxia	6 (12 %)	1 (2 %)	0	−	−	4 (10 %)	3 (8 %)	3 (8 %)	−	−
Cognitive disturbance	4 (8 %)	0	1 (2 %)	−	−	5 (13 %)	1 (3 %)	0	−	−
Concentration impairment	2 (4 %)	1 (2 %)	0	−	−	5 (13 %)	2 (4 %)	0	−	−
Dizziness	4 (8 %)	2 (4 %)	0	−	−	3 (8 %)	1 (3 %)	1 (3 %)	−	−
Dysarthria	0	0	0	−	−	0	1 (3 %)	0	−	−
Dysesthesia	2 (4 %)	0	0	−	−	3 (8 %)	0	0	−	−
Cerebral edema	−	−	1 (2 %)	0	0	−	−	0	0	0
Fatigue	20 (41 %)	3 (6 %)	0	−	−	16 (41 %)	2 (5 %)	0	−	−
Headache	8 (16 %)	0	1 (2 %)	−	−	5 (13 %)	1 (3 %)	1 (3 %)	−	−
Hemiplegia	1 (2 %)	0	0	0	0	0	0	0	0	0
Nausea	2 (4 %)	0	0	−	−	3 (8 %)	1 (3 %)	0	−	−
Paresthesia	2 (4 %)	1 (2 %)	0	−	−	2 (5 %)	0	0	−	−
Seizure	1 (2 %)	0	2 (4 %)	0	0	0	1 (3 %)	0	0	0
Vomiting	4 (8 %)	(8 %)	0	0	0	0	0	0	0	0
Asymptomatic	9 (18 %)					3 (8 %)				
Symptoms unknown	2 (4 %)					1 (3 %)				

Annotations: Toxicity was graded as stated in the Common Terminology Criteria for Adverse Events, version 5.0. ^a^ acute = during treatment until 3 months after fSRT, n = 49. ^b^ chronic= >3 months after fSRT, n = 39.

### 3.5 RICE

After a median time of 3.2 months (Q1-Q3: 1.7–6.2 months) 4 patients (8.1 %) developed RICE. One of them developed moderate symptoms (CTCAE Grade 2) and three experienced life-threatening symptoms with urgent intervention indicated (CTCAE Grade 4). All patients were treated with corticosteroids. Three of them received bevacizumab additionally. In Table 3 further details on RICE are illustrated.

## 4 Discussion

Due to the high morbidity and mortality associated with neurosurgical interventions in BSM, radiotherapy is, in the most cases, the only possible local therapy. The current National Comprehensive Cancer Network (NCCN) guideline recommends SRS for a limited number of BM in patients with good performance status, whereas for patients with multiple BM, WBRT is listed as first treatment of choice [41]. However it should be noted that patients with BSM were mostly excluded from the prospective randomized trials due to their poor prognosis and high local complication rate. For the same reason many centers still perform WBRT for metastases localized in the brain stem due to fear of possible side effects and lack of experience.

A relevant difference compared to older studies is the rise of new systemic therapy options especially IT/TT. More than half of the patients received immunotherapy (61.5 %) during their course of disease and 30 % simultaneously to fSRT. The predominant class of active ingredients were checkpoint inhibitors. It is the subject of current studies to what extent these substances have an intracranial activity [7], [8], [9], [10], [42], [43], [44]. Those patients may have contributed to our favorable results regarding intracranial control and salvage therapy rates. Nevertheless, due to the different tumor entities and thus different systemic therapies, the individual groups are too small to get a significant impact.

In the context of earlier literature, the 1-year-OS of 44.8 % that we found in our cohort compares favorably. Earlier studies showed inferior 1-year-OS about 32.7 % respectively 31 % [31], [32]. Current studies for example Lee et al. described in a narrative review a 1-year-OS of 33 % as well as Chen et al., whereas Nicosia et al. found 1-year-OS of 49.5 %, although the multivariate analyses also showed that concurrent TT was significantly associated with longer OS [24], [25], [29]. The pronounced advances in systemic therapy observed over the past years, especially IT and TT most likely contributed largely to these results [45].

The rate of neurologically related death of 16 % was also superior relative to the published literature (Chen et al. 24 % [29], Nicosia et al.: 30.4 % [25]). A possible reason could be the different RT dose schemes as well as the applied radiation techniques including advanced image guidance. In this study we used a total dose of 30 Gy in six fractions prescribed to the 70 % isodose. This is our department’s standard with the aim of minimizing local side effects at a comparatively high total dose. In previous studies the median dose ranges from 14 Gy to a maximum of 32 Gy in one to five fractions depending on the size of the metastases [25], [29], [31], [32]. For example Trifiletti et al. showed that a RT dose ≥ 16 Gy is a positive predictive factor for longer OS [30].

In our analysis, the 1-year-LC was 84.1 %. This result compares similar to the data of Lee et al. where 86 % of the patients had no local progress after one year as well as with Chen et al. with 86 % local control reported as well [24], [29]. In literature the local control rates of BSM after radiotherapy vary from 81 % to 90 % [25], [30], [31], [32]. These are excellent results which are compatible with the LC of radiosurgery treated BM located anywhere else in the brain [11], [12]. It shows that despite the critical surrounding structures and the associated required dose adjustments/fractionations, excellent LC can still be achieved.

Nevertheless, 69.4 % of the patients suffered from distant CNS progression, with 42.9 % receiving another fSRT/SRS and 22.4 % WBRT. This is also comparable with non-BSM-RT data of Yamamoto et al. where 38 % received salvage SRS [46]. This underscores again that high-resolution brain MRI for CNS surveillance is crucial. However, it should be emphasized that distant failure in local treatments is not surprising.

Overall clinical performance, as described by the KPS, was the most important and statistically significant predictor of improved OS in the current analysis. Patients with a KPS of 90–100 % showed longer OS than patients with an inferior performance status. This finding has been consistent in several previous analyses [32], [47], [48].. Contrary to what we expected, the simultaneous application of immunotherapy was not statistically significant in relation to the OS. This may be related to the comparatively small patient cohort and the fact that the data analysis goes back to times when molecular testing and immunotherapies were not yet standard.

Treatment-related toxicity was rare for this vulnerable localization, and, in most patients, only moderate symptoms CTCAE ≤ grade 2 were diagnosed. Acute grade 3 toxicities occurred in 18.4 % of the patients with ataxia, seizure, a deterioration in cognition and concentration, and headaches. In the case of the latter, it is not always possible to make a clear distinction from side effects of systemic therapy, so there may be an overlap here. Acute grade 3 toxicity of nearly 20 % is relatively high compared to other reports which could be due to the structured and systematic follow-up of our patients which some retrospective studies did not include. After three months of follow-up treatment-related toxicity occurred in about one tenth and was as well mainly ataxia. Studies on SRS of non-BSM also showed that severe toxicity is rare and that the neurological status and the quality of life do not deteriorate significantly [32], [49]. Local therapy can even improve the symptoms [29]. One of the most important and feared side effect of SRS is RICE, nevertheless it is essential to distinguish between RICE and BBD in an interdisciplinary discussion to choose the right therapy accordingly [17]. RICE was rare (8.2 %) but symptomatic in all cases. Compared to the incidence of RICE following SRS reported in the literature our results are favorable. Variability in reported rates of RICE ranges between 5 % and 25 %, depending on the dose, the volume, and the fractionation [50], [51], [52]. The use of a fractionated dose scheme may have contributed to these favorable result. Nevertheless, it should be mentioned that RICE especially around the brainstem is often associated with serious side effects and high mortality [29], [30].

In this retrospective study we showed that fSRT of BSM is a safe and well tolerated therapy method resulting in a high local control rate. A rigorous imaging follow-up for possible local changes and distant CNS progression is essential. Only one fifth of patients required salvage-WBRT.

Limitations of the current analysis includes its retrospective nature and the small number of patients due to the monocentric setting. Therefore previously suggested associations between a combination of fSRT and especially IT/TT and the occurrence of RICE cannot be further assessed [14]. Furthermore, quality of life and neurocognition were not systematically documented and analysed. It must be mentioned that in most cases cause of death was related to a progress of the primary tumor and not due to cerebral progression.

Our analysis is strengthened by its uniform fractionation and dosing scheme and its follow-up performed in a rigorous and homogeneous regimen at a single large tertiary cancer centre.

## 5 Conclusion

This retrospective single-centre study could validate FSRT of BSM as an effective and safe treatment approach with a high local control rate – similar to those reported for lesions in other regions of the brain – and acceptable serious side effects. There is a need for standardized guidelines regarding dose and fractionation of the radiotherapy and therefore the inclusion of patients with BSM to prospective randomized clinical trials is necessary for confirming the results of previous retrospective analyses.

## Declaration of competing interest

The authors declare that they have no known competing financial interests or personal relationships that could have appeared to influence the work reported in this paper.
