# Supplementary material for: Fractionated stereotactic radiotherapy of brainstem metastases – Clinical outcome and prognostic factors
Source: Clin Transl Radiat Oncol. 2024 Nov 21;50:100893. doi: 10.1016/j.ctro.2024.100893 (PMC11621500; doi:10.1016/j.ctro.2024.100893)
Supplement: Supplementary Data 1 [file mmc1.doc]

**Fractionated stereotactic radiotherapy of brainstem metastases – Clinical outcome and prognostic factors.**

**Supplementary Files:**

Supplementary Table 1 Simultaneous Immunotherapy and Targeted Therapy

| **IT/TT simultaneous to fSRT^a^** | | **t½^b^** | **n= 36/45^c^** |
| --- | --- | --- | --- |
| **Checkpoint inhibitors (16/24)** | Nivolumab | 25d | 3 |
|  | Ipilimumab | 15d | 2 |
|  | Pembrolizumab | 22d | 8 |
|  | Atezolizumab | 27d | 3 |
| **HER2 inhibitors (5/24)** | Trastruzumab | 28d | 2 |
|  | Pertuzumab | 18d | 2 |
|  | Lapatinib | 24h | 1 |
| **Alk tyrosine kinase inhibitors (2/24)** | Alectinib | 32h | 1 |
|  | Crizotinib | 42h | 1 |
| **Topoisomerase inhibitors (1/24)** | Sacituzumab | 19h | 1 |

Abbreviation: IT, immunotherapy; TT, targeted therapy; fSRT, fractionated stereotactic radiotherapy; t½; half-life; HER2, human epidermal growth factor receptor 2; ALK, anaplastic lymphoma kinase. ^a^ Any dose of immunotherapies given within 14 days before or after fSRT. ^b^ Information about half-life was extracted from the clinic intern drug information service (AID). ^c^ Of the 20 patients that received simultaneous IT/TT with fSRT four patients had treatment with two different drugs at the same time.

Supplementary Table 2 Simultaneous Chemotherapy

| **CTx, simultaneous to fSRT^a^** | | n= 11/17^b^ |
| --- | --- | --- |
| **Platinum analogues (6/17)** | Carboplatin | 5 |
|  | Cisplatin | 1 |
| **Taxan (4/17)** | Paclitaxel | 4 |
|  | Docetaxel | 1 |
| **Pyrimidine antagonists (3/17)** | Capecitabine | 2 |
|  | Gemcitabine | 1 |
| **Vinca Alkaloid (2/17)** | Vinorelbine | 1 |
| **Others (2/17)** | Polychemotherapy regimes | 2 |

Abbreviation: CTx, chemotherapy; fSRT, fractionated stereotactic radiotherapy. ^a^ Any dose of chemotherapies given within 14 days before or after fSRT. ^b^ Of the 11 patients that received simultaneous CTx with fSRT six patients had treatment with two different drugs at the same time.
